# A rural teledentistry care experience: a geriatric approach to assessing oral health status and treatment needs in older adults from a Mapuche community in Chile

**DOI:** 10.3389/fpubh.2024.1356622

**Published:** 2024-06-05

**Authors:** Víctor Beltrán, Fernanda Muñoz-Sepúlveda, Claudia Acevedo, Pablo Navarro, Bernardo Venegas, Catalina Salgado, Pablo Uribe, Wilfried Engelke

**Affiliations:** ^1^Clinical Investigation and Dental Innovation Center (CIDIC), Dental School and Center for Translational Medicine (CEMT-BIOREN), Universidad de La Frontera, Temuco, Chile; ^2^Interuniversity Center for Healthy Aging (CIES), Santiago, Chile; ^3^Program of Master in Dental Science, Dental School, Universidad de La Frontera, Temuco, Chile; ^4^Research Center for Dental Sciences (CICO), Dental School, Universidad de La Frontera, Temuco, Chile; ^5^Faculty of Health Sciences, Universidad Autónoma de Chile, Temuco, Chile; ^6^Stomatology Department, Faculty of Dentistry, Universidad de Talca, Talca, Chile; ^7^Dental School, Universidad de La Frontera, Temuco, Chile; ^8^Faculty of Medicine, Georg-August University of Göttingen, Göttingen, Germany

**Keywords:** oral health, indigenous, oral disease burdens, teledentistry, aging, inequalities, geriatric assessment, older adults

## Abstract

**Background:**

Limited attention has been given to oral health challenges faced by older Indigenous populations, especially in rural settings, where disparities exist. This study aims to assess oral health in a rural Mapuche community in southern Chile, utilizing geriatric technology support, and exploring the connection between geriatric health and oral well-being to fill a gap in this context.

**Methods:**

A cross-sectional study was conducted involving 76 independent older adults from a rural Mapuche community who required dental care. Assessments were in a remote care setting gathering extensive data including comprehensive geriatric assessments, medical and dental conditions using a geriatric teledentistry platform (TEGO^®^). Statistical analysis involved descriptive analysis, logistic regression, and both multiple correspondence analysis and k-means cluster analysis.

**Results:**

The sample comprised individuals with limited formal education and a high degree of vulnerability. Geriatric assessments unveiled cognitive deterioration, frailty, depression risk, and multimorbidity. A distribution of the DMFT index, number of remaining teeth, number of occluding pairs, number of teeth with restorative needs and other relevant clinical findings was conducted based on sociodemographic, and medical-geriatric-dental characteristics, and additionally, a Multinomial Logistic Regression Analysis of Dentition Variables in Relation to Geriatric Assessments was performed. The dental burden was substantial, with an average DMFT index of 25.96 (SD 4.38), high prevalence of non-functional dentition (89.3%), periodontal disease (83%), xerostomia (63.2%) and oral mucosal lesions (31.5%). Age, lower education, depression, daily medication number and sugary consumption frequency were associated with a decreased average number of teeth (*p* < 0.05). Multiple correspondence analysis and k-means cluster analysis identified 4 clusters, with the edentulous and functional dentition groups being the most distinct.

**Conclusion:**

This study uncovers a substantial dental burden and intricate medical-geriatric conditions interlinked among Indigenous older adults in a rural Chilean Mapuche community. The implementation of a geriatric technological ecosystem in the community enabled the resolution of less complex oral health issues and facilitated remote consultations with specialists, reducing the necessity for travel to health centers. This underscores the need for innovative dental public health initiatives to address health disparities and improve the overall well-being of older Indigenous adults.

## 1 Introduction

Numerous global references have focused on enhancing the oral health of older adults (1–5). However, limited attention has been directed toward the dual challenge faced by older Indigenous populations, stemming from restricted dental access and age-related concerns (6), which is further worsen by the disparities between Indigenous and non-Indigenous oral health outcomes (7–10). Moreover, in the present day, Indigenous communities are among the most disadvantaged globally (11, 12), facing an inequitable burden of health disparities, which differ across continents and are primarily linked to a mix of factors, such as socioeconomic disadvantages, communication barriers, transportation limitations, inadequate and inconsistent funding for community health services, limited access to culturally suitable healthcare, summed to unique social obstacles such as systemic racism, colonialism/neocolonialism, globalization, migration, transgenerational loss of language, land and culture, and even climate change (8, 13–16). Given potential disparities in funding for oral and general healthcare, it is imperative to thoughtfully consider the potential greater challenge of addressing oral health inequalities compared to general health inequalities (17).

Furthermore, rural communities face challenges associated with aging, marked by the departure of working-age individuals to urban areas and the influx of retirees from cities, intensifying the demand for healthcare (18). Coupled with these demographic shifts, rural health settings grapple with significative administrative, quality control, and logistical hurdles, including a shortage of trained personnel and limited access to specialists (19). Globally, the utilization of health information and communication technologies (e-health) has proven instrumental in overcoming geographical barriers, emerging as a viable option for providing oral care services in rural and remote areas (20). These technologies have positively impacted general health condition management, contributing to enhanced healthcare access, improved quality of life, and increased social support within Indigenous populations (21, 22). Despite these benefits, Indigenous e-health research remains underexplored and underfunded, predominantly focusing on younger Indigenous populations in developed countries (21–23).

Within the framework of human rights and sustainable development, initiatives have been undertaken to expedite endeavors aimed at enhancing Indigenous populations’ access to healthcare as a means of promoting well-being, justice, and human dignity (24–26). Nonetheless, achieving this goal requires the development of scientific evidence regarding the epidemiological profiles of this population (24) and the formulation of community-tailored programs. Based on global experiences, it has been emphasized that strategies such as community health programs, student-led healthcare services, outreach initiatives, mobile clinics, and e-health could play a pivotal role in achieving this goal (27).

In that context, our group has made efforts in implementing an innovative teleodontology strategy for older adult residents of rural areas in Chile, with a special focus on the Mapuche community, which constitutes the most numerous Indigenous group in Chile, reaching around one million people in the central and southern regions of the country. 26% of the population in the La Araucanía Region, over 14 years old, identifies as such.

The absence of comprehensive epidemiological data among Latin American Indigenous groups represents a significant hurdle to advancing tailored oral health awareness and preventive initiatives (9). Moreover, a notable gap exists in initiatives addressing the distinctive requirements of older Indigenous individuals residing in rural settings, coupled with a scarcity of studies exploring the utilization of e-health, particularly e-oral healthcare, within this population, to the best of our knowledge. In this context, this study aims to assess the oral health status and treatment requirements of older adults within a rural Mapuche community in the La Araucania region of Chile, leveraging support from a geriatric technological ecosystem. Additionally, it seeks to explore the association between the sociomedical geriatric state and oral health, addressing an unaddressed area within the context of this Indigenous community.

## 2 Materials and methods

### 2.1 Ethical considerations

This cross-sectional study was developed as part of a Presidential Grant Project called Clinical Center for Innovation in Oral Health of the Older Adult in La Araucania region (Exempt Resolution No. 472). The main objective of this project is to implement interventions aimed at preventing and treating oral diseases, along with initiatives to enhance oral health knowledge, behaviors, and oral health self-efficacy. Ethical approval for conducting this project was granted by the Universidad de La Frontera Ethics Committee, decision 109/22. All the participants agreed to participate by signing an informed consent and their confidentiality was granted by anonymization of personal information prior to data treatment.

### 2.2 Participants and setting

In Chile, 12.8% of the population belongs to Indigenous communities, Mapuche people are the most prominent group, making up 32.82% of the population in the La Araucania region (28). The traditional Mapuche territory spans from the Bio-Bio River to Chiloe Island, encompassing diverse territorial identities that diverge from official state boundaries; each holds its distinct socio-cultural, spiritual rationale, and medical-religious framework (29, 30). The Makewe-Pelale sector belongs to the Wenteche territory and is in the La Araucania region, specifically in the municipalities of Padre las Casas and Freire. It consists of approximately 80 Mapuche communities with an estimated population of 10.000 people; the residents of this sector are mainly small-scale farmers, with an average of only 1.5 hectares per capital (29). This rural sector does not have access to fluoridated water.

The first approach to the Makewe-Pelale community was through a social anthropologist (A.H) with many years of collaborating with the community. Then, a meeting was made with the Werkén, spokesperson for various Mapuche organizations (31), and the Trapilhue leaders to explain the project, learn about their perspectives and perceived needs. The community through its council designated an intercultural facilitator of the community (E.Q) who spoke Mapuzugun (the language spoken by the Mapuche community), to be in charge of recruiting and enrolling the participants. Due to the scarce statistical records in this geographic area and the poor connectivity, the recruitment was through a convenience sampling, in which the intercultural facilitator went house to house to recruit participants. As in Beltrán et al. (32), inclusion criteria considered older adults (60 years and older) requiring an emergency, priority, or dental check-ups. Requirements to provide dental care in a remote setting were specified as follows: patients with chronic diseases must be under pharmacological treatment according to medical indications; patients must be capable of receiving verbal instructions and have sufficient mobility to sit in a portable dental chair. If they met the selection criteria and agreed through informed consent, they were scheduled to receive dental care in an Experimental Campus of Universidad de La Frontera located in this area.

Patients were enrolled face-to-face by the facilitator trained for it. Data considered for the patient’s registration included socio-demographic information such as their full name, national ID number, date of birth, contact phone, address, social register household (RSH) (33), ancestry, educational level, occupation, and social support. Barthel Index for Activities of Daily Living (34) was applied. All the data and instruments were loaded on a web platform named Geriatric Dental Specialties Tele platform (TEGO by its acronym in Spanish: “Tele Plataforma de Especialidades Geriátrico Odontológicas”) (32). This platform constitutes a technological ecosystem for geriatric clinical care, incorporating an electronic dental assessment record, 3D models, and facilitating teleconsultations with specialists.

The setting was a classroom prepared in workstations for the integral assessment and treatment of the older adults, four fully portable dental equipment and a portable dental x-ray equipment were used.

The inclusion criteria considered the older adult population (over 60 years old) with a dental emergency or requiring some kind of priority dental care. The requirements for providing dental care in a mobile clinic were specified as follows: sufficient mobility to access a dental chair in a mobile clinic; patients with chronic diseases must be under pharmacological treatment according to medical indications; patients must be capable of receiving verbal instructions and must complete a triage before the provision of dental care. The exclusion criteria apply to patients with vital emergencies (e.g., anaphylactic crisis) that must be immediately assessed by a physician and require urgent care.

### 2.3 Data collection

The examinations were conducted by two dental students completing their final year of internship, who were trained and supervised by two dentists with extensive clinical experience in the field (C.A and F.M-S). Before the examinations, the examiners were trained on the study protocol and diagnostic criteria using a PowerPoint presentation. Inter- and intra-examiner agreement (kappa values) was calculated. For the inter-examiner agreement, the students examined a total of 445 dental surfaces across six patients, while for the intra-examiner agreement, they re-examined 245 dental surfaces 1 week later. The results showed substantial inter-examiner agreement (*κ* value = 0.66) and substantial to almost perfect intra-examiner agreement (*κ* values of 0.78 and 0.85, respectively).

Clinical data were recorded following the criteria and recommendations from well-established methods (35, 36). The TEGO platform integrates three anamnesis modules for integral asses of older adults, allowing the evaluation of:

#### 2.3.1 Medico-geriatric data

- Cognitive state: The assessment was made through the Mini Mental state examination (MMSE) (37) or the Short Portable Mental Status Questionnaire (SPMSQ) (38), according to educational level. Cognitive deterioration was defined with <25 points in MMSE or > 3 points in SPMSQ.
- Depression: The shortened Yesavage geriatric depression scale was evaluated (39).
- Frailty: Fried frailty phenotype (40) was applied.
- Nutritional status: Was calculated through Body Mass Index using the values for weight and height. A BMI ≥ 25 was considered overweight.
- Multimorbidity: Multimorbidity has been defined as the presence of two or more comorbidities (41), and was obtained from participants’ reports.
- Number of daily medications: Medication consumption was obtained from the participants reports. Polypharmacy was defined as the consumption of five or more medication (42).
- History of falls in the past year, obtained from the participant report.

#### 2.3.2 Dental-geriatric data

**Figure 1:**
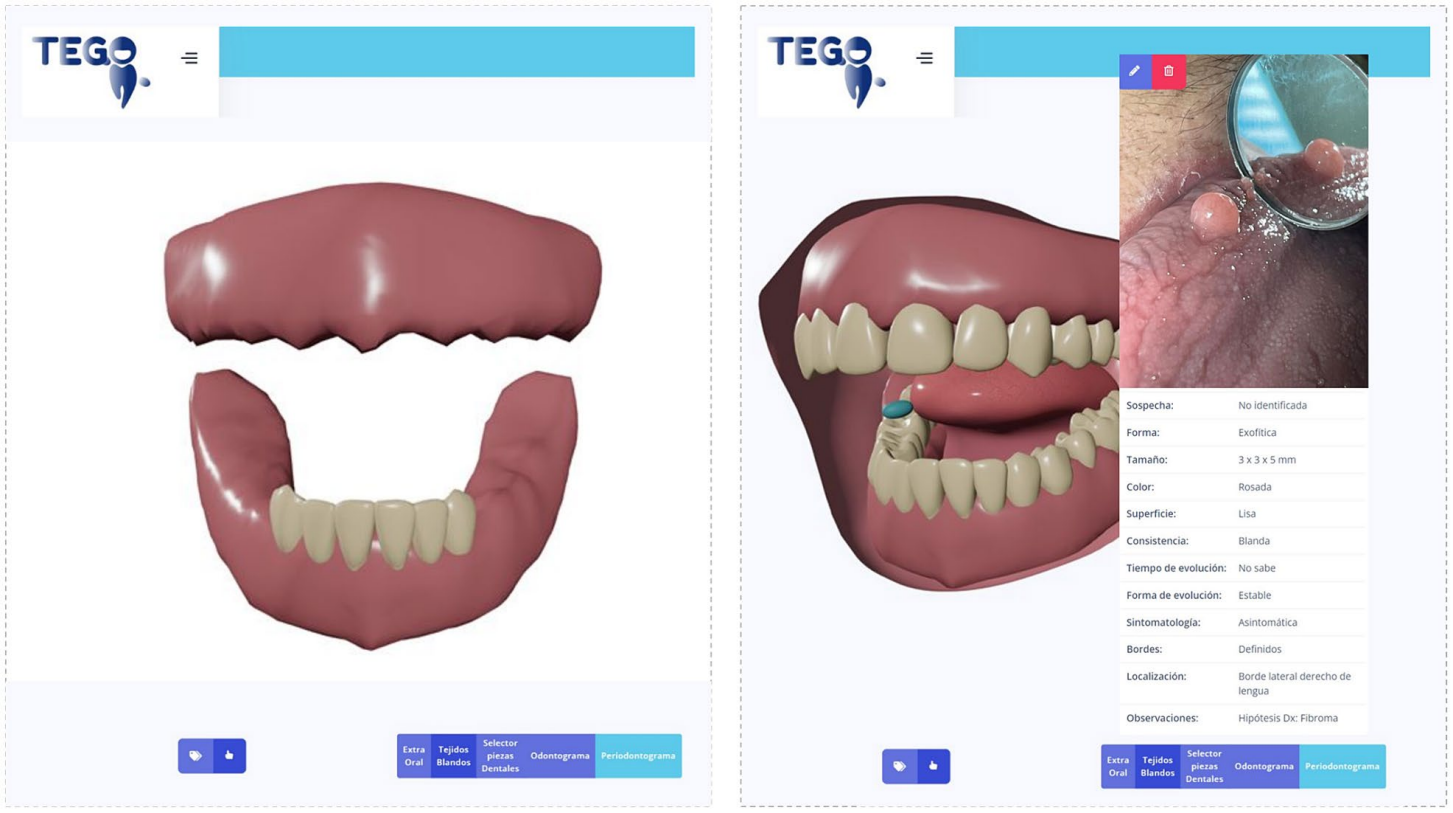
Platform module interface, 3D standardized dental model, and 3D standardized intraoral and soft tissues examination. On the left side, the three-dimensional modeling depicts the most prevalent occlusion in the studied sample, using a standardized 3D phantom contained within the TEGO platform. On the right, there is the 3D phantom developed on the TEGO platform, which allows for generating a general representation of the intraoral soft tissues (mucous membranes and tongue), labeled in this case with a presumptive diagnosis of Fibroma; the interface enables remote evaluation and communication with an oral pathology specialist, through teleconsultation using the TEGO platform.

- Lifestyle-related characteristics: A first interview was conducted for the assessment of habits such as brushing frequency, use of dental floss, cariogenic diet consumption and unhealthy habits. The questions surveyed were: How many times per day do you brush your teeth: never/once a day/twice a day/ three times a day/more than three times a day?; Do you use dental floss?: yes/ no; concerning the consumption of a cariogenic diet, the questions were as follows: Do you consume sugary foods and/or drinks (such as candies, cookies, cakes, sweet cakes, soda or sugary juices, coffee, tea, mate or milk with sugar) between meals: yes/no?. If the answer was yes, a question of the number of times in the day was added. Smoking habit was surveyed with the following question Do you smoke: yes/no?
- Last visit to dental care, obtained from the participant report.
- Swallowing disorders: The EAT-10 tool (43) was applied for the assessment of swallowing disorders. If the score was three or more, it was considered positive (44).
- Xerostomia assessment: It was surveyed the question Do you feel your mouth dry: yes/no?
- Dental Examination: Dental assessments were conducted employing dental mirrors and a periodontal probe, encompassing evaluations of teeth in both wet and air-dried states while considering both the coronal and root surfaces. Detailed explanations of the ICDAS-II scores for diverse levels of dental caries on all surfaces were previously provided (45). The computation of DMFT indices followed this approach: ICDAS scores 0 to 3 were considered as D = 0 (sound); scores 4 to 6 were interpreted as D = 1 (requiring restoration); missing teeth were designated as M = 1; and restored teeth without caries were attributed *F* = 1. Furthermore, the presence of non-carious tooth surface loss was also evaluated. The oral health status of the teeth was recorded in the platform’s odontogram section.
- Periodontal assessment: was made with UNC-15 probe (Hu-Friedy, Chicago, Illinois, United States) performing a full-mouth recording on the SEPA periodontogram (46), which includes periodontal measurement at six sites per tooth. Gingival bleeding was evaluated after probing all teeth with an oral mirror. Periodontitis was defined when interdental CAL ≥ 2 non-adjacent teeth, or Buccal or Oral CAL ≥ 3 mm with PPD > 3 mm is detectable at ≥2 teeth (47); gingivitis was defined as ≥10% bleeding sites with probing depths ≤3 mm; and gingival health was defined as <10% bleeding sites, with probing depths ≤3 mm (48).
- Missing teeth and number of occluded pairs: The number of natural teeth and tooth condition was assessed using the WHO criteria (35). The number of natural teeth was categorized into four groups: Total tooth loss (0 teeth), severe tooth loss (1–9 teeth), 10–20 teeth and functional dentition (21 or more teeth) (49, 50). The state of the teeth was uploaded in a novel 3D standardized model in TEGO (51). The analysis of the 3D model was used to ascertain the number of occluded pairs, where opposing pairs of maxillary and mandibular teeth were considered as a single occluded pair.
- Oral mucosal lesions (OML): Following WHO guidelines (35), a comprehensive examination of oral mucosa and soft tissues, involving both visual inspection of the perioral area and a systematic assessment of the oral mucosa, was conducted. Detected OML were documented on the platform with traditional descriptions (size, color. Limits, symptomatology, type of surface, consistency, localization, and evolution), 3D digital representations (utilizing an extra and intraoral 3D phantom), and clinical images (52) (see Figure 1). Specialists received timely information through auto-generated emails and provided responses directly through the platform. The assessment aimed to identify the presence or absence of OML, offering an initial clinical diagnosis and classification, including denture related lesion, infectious conditions, oral potentially malignant disorder, tumors and tumor-like lesions, pigmentations, immune-mediated conditions, physical and chemical injuries, and vascular malformations (53–56), while also noting the affected surfaces. Additionally, the oral pathology specialist provided treatment suggestions or follow-up recommendations.
- Prosthetic Evaluation: The assessment of dental prostheses involved evaluating rehabilitation requirements, the use of prostheses, and examining the state of appliances employed by individuals. During the clinical examination, a thorough analysis was conducted, encompassing defects, stability, retention, and the overall integrity of the appliance, all based on established criteria (57, 58).

**Figure 2:**
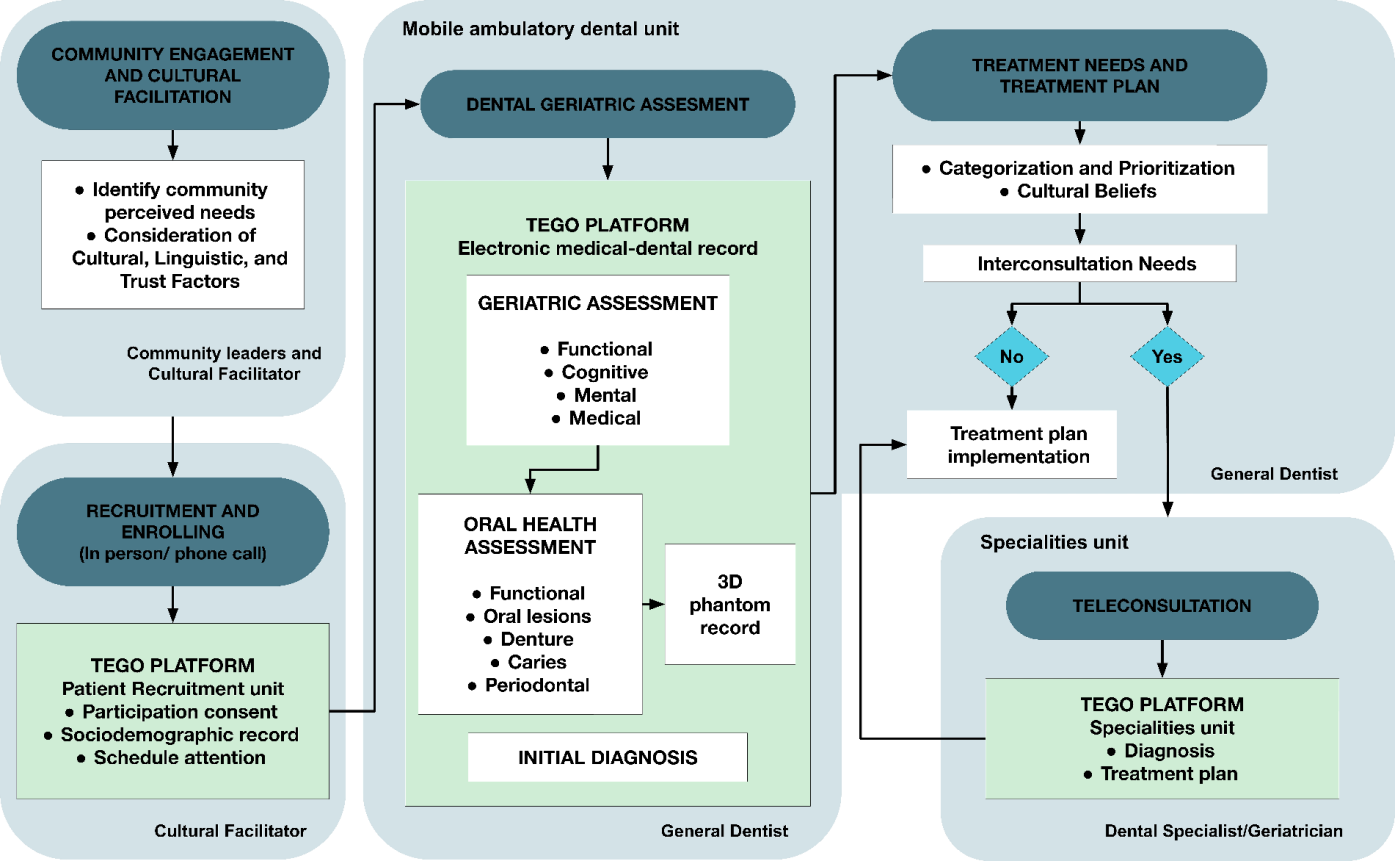
TEGO comprehensive care pathway. Operational flowchart of the attention and platform use workflow to support dental-medical care for older adults in a rural Mapuche community. Includes the Macroprocess description, the Units, and people involved in its development.

Figure 2 depicts the TEGO comprehensive care pathway tailored for assessing and treating oral health within older adults of a rural Mapuche community. This pathway encompasses a holistic approach integrating teledentistry alongside conventional methods to address the unique oral health needs of this demographic. From initial screening to treatment plan implementation, each step was meticulously designed to consider cultural sensitivities, ensure patient-centered care, and leverage teledentistry for enhanced accessibility and outreach.

### 2.4 Statistical analysis

The descriptive analysis encompassed frequencies, means, standard deviations, and confidence intervals for the investigated parameters. Associations between outcomes and explanatory factors were investigated employing cross-tabulation alongside Pearson χ2 and Fisher’s exact tests. Additionally, a Multinomial logistic regression analysis was conducted to examine the relationship between tooth loss variables and the medico-geriatric state of the participants. Multiple correspondence analysis and K-means cluster analysis was performed to gain insights into population profiles in concerning the number of present teeth. The data collection was recorded in a Microsoft Office Excel spreadsheet, a descriptive analysis of the data was carried out for which the mean and its respective standard deviation and frequency distribution table were determined, the Kolgomorov-Smirnov normality test was carried out., Pearson’s Chi-Square test and Fisher’s Exact test, Kappa index as a measure of agreement, multivariate analysis through: Multinomial Logistic Regression, Multiple Correspondence Analysis and k-means Cluster Analysis. The SPSS statistical program (Statistics for Windows, version 23.0, IBM) was used to analyze the data.

Statistical significance was established at *p*-values <0.05. All statistical analyses were conducted employing SPSS software, version 23.

## 3 Results

At the conclusion of the survey, data were collected from a total of 76 participants. The participants had an average age of 70.55 years (SD 7.33), with 51.3% of them being women. Half of the older adults (51.4%) fell within the age range of 65 to 75 years. Most participants (78.9%) had 8 ears or less of formal education and were classified in the most vulnerable level of the RSH (98.4%). Almost half of the respondents considered themselves active by engaging in agriculture work (47.2%).

### 3.1 Geriatric assessment

In the mental and emotional evaluation, 20 participants (26.3%) had cognitive deterioration, and 32.9% were at risk of depression. The participants had a poor history of medical control, with 65.5% being overweight, 48.7% having uncontrolled blood pressure, and 18.4% having hyperglycemia. The average number of chronic diseases per participant was 1.58 (95% CI 1.27; 1.88), and 48.7% of them had multimorbidity. The daily consumption of medications varied from 0 to 10, with an average of 2.17 (95% CI 1.68; 2.66). As for functional status, almost all participants were entirely independent (93.4%). In terms of physical frailty, only one participant was classified as robust, while 51.3% exhibited frailty. Additionally, 39.5% of participants reported a history of falls in the past year.

### 3.2 Lifestyle characteristics

Regarding oral health behaviors, a considerable proportion (90.8%) of participants engaged in regular tooth brushing. The majority brushed their teeth two (37.8%) or three (32.4%) times a day and 26.2% flossed their tooth. The consumption of sugary items between meals was present in 28.9% of the participants, with an average of around two instances per day (mean 1.87; 95% CI 1.49; 2.23). Tobacco usage was observed in merely six individuals (7.9%). Additionally, 47.4% of participants had undergone a dental visit within the past year.

### 3.3 Dental caries experience

All the participants had a history of dental caries, as measured by the DMFT index, ranging from 13 to 32, with a mean of 25.96 (95% CI 24.88; 27.05) affected teeth. They had an average of 1.09 healthy teeth (95% CI 0.49; 1.69), 2.52 coronal caries (95% CI 1.89; 3.14), and 0.36 root caries (95% CI 0.16; 0.56) per teeth. Additionally, they had an average of 3.75 restorations (95% CI 2.74; 4.76) and six non-carious lesions (95% CI 5.12; 6.87).

### 3.4 Tooth loss

Concerning dentition status, the mean number of present teeth was 9.67 (95% CI 7.95; 11.39), 13 participants (17.1%) were fully edentulous, and only 10.7% of participants had functional dentition. Among those with partial dentition, the mean number of occluding pairs was 2.33 (95% CI 1.52; 3.15). The TEGO platform enabled the three-dimensional modeling of the most prevalent occlusal characteristics in the studied sample, with the most frequent findings being total maxillary edentulism and preservation of the lower anterior sextant (Figure 1).

### 3.5 Periodontal health

The prevalence of periodontal diseases was found to be substantial. Among the participants, 6.7% exhibited healthy periodontal conditions, whereas 10% presented with gingivitis. Notably, periodontitis was prevalent in 83.3% of participants.

### 3.6 Other oral manifestations

A total of 63.2% of participants self-reported experiencing symptoms of dry mouth, while nearly one in five (19.7%) reported having swallowing difficulties.

Both xerostomia and swallowing difficulty were found to exhibit significant correlations with each other (*p* < 0.05). Furthermore, they demonstrated correlations with multimorbidity (*p* < 0.05) and depression (*p* < 0.01). Additionally, swallowing disorders were also correlated with the number of medications consumed (*p* < 0.01).

30 oral mucosal lesions were found, which manifested in almost one-third of the participants (*n* = 24, 31.58%). Table 1 illustrates the distribution of all mucosal disorders observed in the sample. The most frequent anatomical location was Palate (33.3%), followed by Lower lip (26.7%). For 26.3% of the participants, a teleconsultation to oral medicine was conducted through the TEGO platform (Figure 1). The majority of cases were managed with conventional dental treatment, pharmacological management, or control and education. Biopsies were required for six cases.

**Table 1:** Prevalence of oral mucosal lesions.

Classification	Oral mucosal lesion	n	%
Denture related lesion	Denture stomatitis	6	20.00
	Denture-associated inflammatory fibrous hyperplasia (Epulis Fissuratum)	3	10.00
Oral potentially malignant disorder	Actinic cheilitis	2	6.67
	Leucoplakia	2	6.67
	Erythroplakia	1	3.33
Vascular lesion	Vascular malformation	5	16.67
Tumors and tumor-like lesions	Irritation fibroma	4	13.33
Pigmentations	Solitary pigmented lesions	1	3.33
	Multiple-pigmented lesions	1	3.33
Immune-mediated conditions	Benign migratory glossitis	1	3.33
	Recurrent aphthous stomatitis	1	3.33
Physical and chemical injuries	Traumatic ulcerations	1	3.33
	Traumatic erythema	1	3.33

### 3.7 Treatment needs

The average number of treatments performed was 3.79 [95% CI 3.57; 4.01]. Among the participants, 44.7% required fillings, with a mean of 2.14 [95% CI 1.52; 2.77] restorative treatments per tooth. This need for restorative treatment increased among the 75 years and older age group, as shown in Table 2. Extraction was indicated in 11.9% of participants. Almost all participants received preventive and promotional treatments, including oral health promotion (97.4%), scaling and dental prophylaxis (76.3%), and approximately one in five (19.7%) underwent fluoride varnish treatment.

**Table 2:** Distribution of DMFT index, number of remaining teeth, number of occluding pairs, and number of teeth with restorative needs based on sociodemographic and medical-geriatric-dental characteristics.

	Variables	*n* = 76	DMFT Mean (SD)	Remaining teeth Mean (SD)	Occluding pairs Mean (SD)	Restorative needs Mean (SD)
Sex	Female	39	26.63(3.25)	11.20 (6.19)	2.20 (2.81)	1.63 (1.88)
	Male	37	25.65 (4.76)	12.72 (6.83)	2.34 (3.30)	2.76 (2.92)
Age groups	60–64 years	21	25.55 (4.11)	12.65 (6.51)	2.25 (2.65)	2.05 (2.35)
	65–74 years	39	26.47(4.34)	11.10 (6.68)	2.03 (3.33)	1.93 (2.42)
	75 years and more	16	26.44 (3.13)	13.22 (6.20)	3.11 (2.98)	3.33 (3.00)
Education level	8 or fewer years of education	57	26.54 (3.87)	11.05 (6.14)	1.80 (2.46)	1.83 (2.29)
	9 or more years	19	25.28 (4.46)	14.00 (7.03)	3.33 (3.93)	3.00 (2.81)
Currently employed	Yes	34	25.63 (3.38)	12.30 (5.57)	2.22 (2.62)	2.30 (2.43)
	No	42	26.59 (4.56)	11.66 (7.28)	2.31 (3.38)	2.09 (2.58)
Frailty phenotype	Pre-frail	36	26.43 (4.12)	12.70 (6.77)	2.93 (3.24)	2.67 (2.82)
	Frail	39	25.64 (3.95)	11.43 (6.20)	1.60 (2.74)	1.75 (2.05)
Cognitive decline	Yes	20	26.61 (3.55)	10.61 (4.52)	1.23 (2.01)	2.08 (2.46)
	No	56	26.02 (4.22)	12.33 (6.96)	2.57 (3.22)	2.22 (2.53)
Depression	Yes	25	27.21 (4.32)	9.43 (6.34)	1.57 (2.47)	1.70 (2.09)
	No	51	25.82 (3.97)	12.73 (6.42)	2.49 (3.18)	2.33 (2.61)
Multimorbidity	Yes	37	25.64 (4.49)	11.56 (6.77)	2.16 (3.05)	1.32 (2.06)
	No	39	26.53 (3.73)	12.23 (6.39)	2.35 (3.06)	2.82 (2.62)
Polypharmacy	Yes	27	27.518 (5.13)	8.15 (7.75)	1.48 (2.47)	1.39 (2.09)
	No	49	27.42 (4.04)	9.7 (7.14)	1.60 (2.86)	2.22 (2.33)
Dental care received during the past year	Yes	36	26.38 (4.07)	13.31 (6.45)	3.24 (3.16)	2.86 (2.56)
	No	40	25.93 (4.10)	10.63 (6.39)	1.33 (2.63)	1.53 (2.28)
Dry mouth	Yes	48	26.34 (4.23)	11.13 (6.67)	2.00 (3.10)	2.34 (2.61)
	No	28	25.81 (3.80)	13.43 (6.07)	2.76 (2.91)	1.90 (2.30)
Periodontitis	Yes	50	26.14 (4.03)	12.02 (6.62)	2.47 (3.14)	2.18 (2.54)
	No	10	26.20 (4.42)	11.69 (6.26)	1.30 (2.36)	2.20 (2.35)

Prosthetics were used by 76.3% of the participants, with 26.8% of individuals seeking consultations for issues related to prosthetics. Most dentures exhibited inadequacies, primarily attributed to cracks, fractures, or poor fit, needing repairs or replacements in a substantial proportion of the participants (85.5%). Relining and repair of the prosthetics were undertaken for 45% of the participants.

The need for dental prosthetics was significantly associated with overweight (*p* < 0.05) and sugar intake between meals (*p* < 0.05). The number of teeth requiring restoration showed a significant relationship with hyperglycemia (*p* < 0.05). Extractions were significantly correlated with multimorbidity (*p* < 0.01).

### 3.8 Sociomedical geriatric state and tooth loss

A multinomial logistic regression analysis was conducted to explore the relationship between geriatric health variables, categorized into four dimensions (social, functional, medical, and dental), and tooth loss-related variables, specifically the number of remaining teeth and the number of occluding pairs. The statistically significant models identified allow for predictions to be made about the studied variables by considering their geriatric characteristics. As illustrated in Table 3, the analysis of social factors reveals that individuals aged 65 and older with lower educational attainment (8 years or less) tend to exhibit a reduction in the number of teeth, with an estimated average decrease of 9.07 teeth. In the mental health assessment, the presence of depression and cognitive decline are associated with a decrease in the average number of remaining teeth and occluding pairs, respectively. Furthermore, a notable disparity emerges in the number of teeth among individuals who consume substantial amounts of sugars between meals, with an estimated difference of approximately 17 teeth.

**Table 3:** Multinomial logistic regression analysis of dentition variables in relation to geriatric assessments.

Social assessment				
Age categorization	8 or fewer years of education	Number of remaining teeth^a^	Number of occluding pairs^b^	*p*-value
60–64 years	Yes	11.16	-	0.006
	No	16.34	-	
≥65 years	Yes	7.27	-	
	No	12.44	-	
-	Yes	-	1.37	0.017
	No	-	3.16	
Functional assessment				
Frailty phenotype		Number of remaining teeth	Number of occluding pairs^c^	*p*-value
Yes		-	1.15	0.032
No		-	2.56	
Mental assessment				
Depression	Cognitive decline	Number of remaining teeth^d^	Number of occluding pairs^e^	*p*-value
Yes	-	6.00	-	0.002
No	-	11.50	-	
-	Yes	-	0.80	0.048
-	No	-	2.28	
Medical assessment				
Number of daily medications	Uncontrolled blood pressure	Number of remaining teeth^f^	Number of occluding pairs^g^	*p*-value
0	-	11.68	-	0.022
1	-	10.76	-	
2	-	9.85	-	
5	-	7.11	-	
7	-	5.28	-	
10	-	2.55		
-	Yes	-	1.05	0.019
	No	-	2.58	
Dental assessment				
Frequency of sugary intake between meals		Number of remaining teeth^h^	Number of occluding pairs	*p*-value
0		18.02	-	0.010
1		13.69	-	
2		9.36	-	
3		5.04	-	
4		0.71	-	

^a^Y = 12.44 + 3.89*Age categorization - 5.18*Educational level; ^b^Y = 3.16–1.78*Educational level; ^c^Y = 2.56–1.40*Frailty; ^d^Y = 11.50–5.50*Depression; ^e^Y = 2.28–1.48*Cognitive decline; ^f^Y = 11.68–0.91*N° of medications; ^g^Y= =2.58–1.52*Uncontrolled pressure; ^h^Y = 18.02–4.33*N° sugary intakes.

A final multinomial regression analysis was conducted, incorporating variables that had been identified as statistically significant within each dimension. This culminated in the development of a conclusive model, capable of predicting the key variables pertaining to comprehensive geriatric assessment that warrant evaluation to ascertain the count of remaining teeth. These variables included age categorization, education level, frailty, cognitive decline, depression, the number of daily medications, and the frequency of sugar intake between meals $\begin{array}{l} (\Upsilon =16.00+0.96*\mathrm{AgeCategorization}-3.71*\mathrm{Education}\\ -1.32*\mathrm{Frailty}-0.59*\mathrm{CognitiveDecline}-3.14*\mathrm{Depression}\\ -0.65*\mathrm{Medications}-0.85*\mathrm{FrequencySugarIntake};p=0.013)\text{.} \end{array}$ Additionally, age categorization, uncontrolled blood pressure, and the frequency of sugar intake between meals emerged as pertinent factors for determining the number of occluding pairs $\begin{array}{l} (\Upsilon =2.66+2.09*\mathrm{Age}\;\mathrm{Categorization}-1.19*\mathrm{Uncontrolled}\;\\ \mathrm{Blood}\;\mathrm{Pressure}-0.49*\mathrm{Frequency}\;\mathrm{Sugar}\;\mathrm{Intake};p=0.013)\text{.} \end{array}$

**Figure 3:**
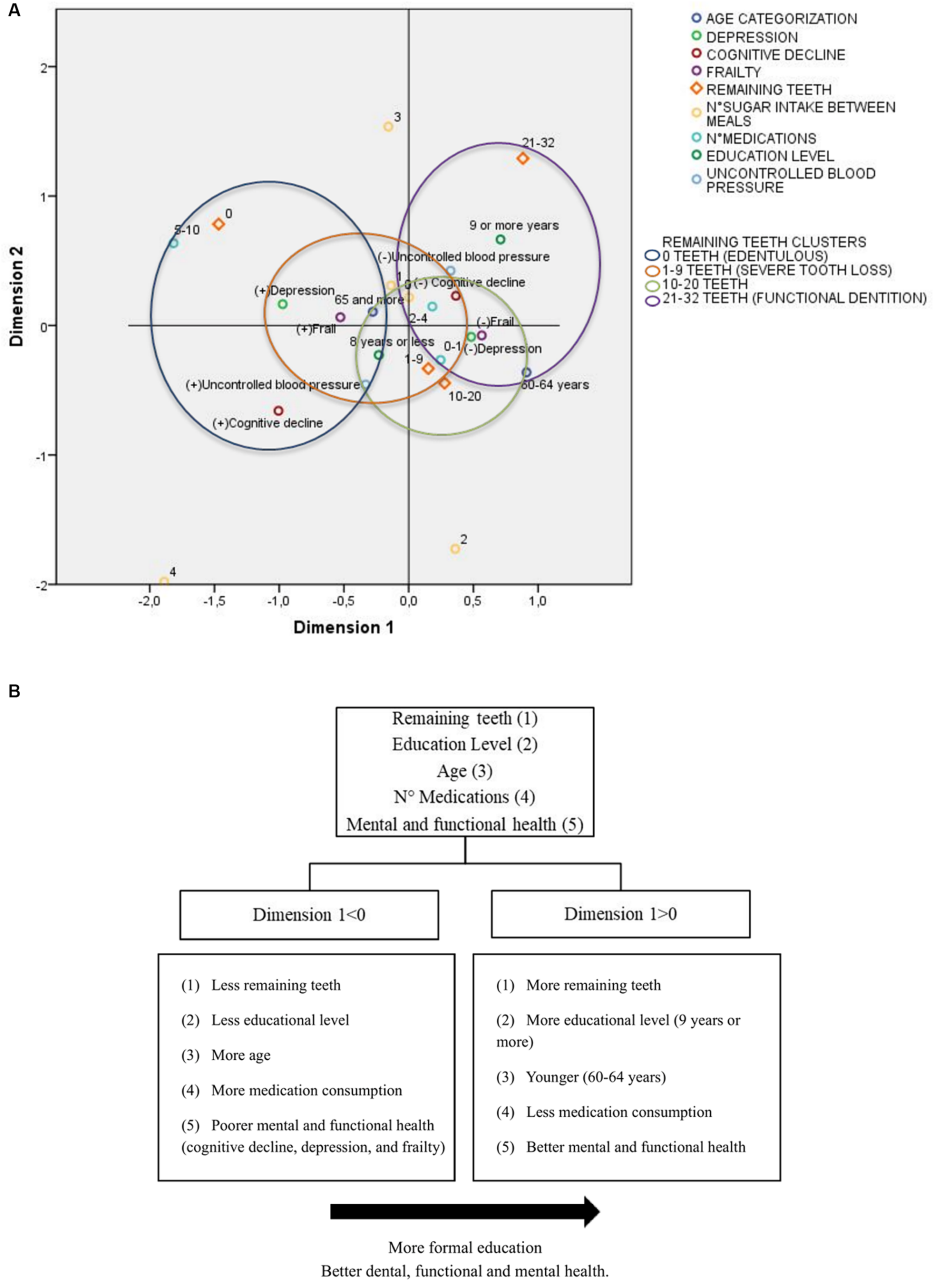
Profiles of remaining teeth using multiple correspondence analysis and k-means cluster analysis, grouped by geriatric assessment characteristics. **(A)** MCA dimensions discrimination measures grouped in clusters by the number of remaining teeth (0, 1–9, 10–20, and 21–32) by k- mean cluster analysis. **(B)** Positive and negative centroid coordinates for dimension 1.

In the multiple correspondence analysis (MCA), a two-dimensional model was obtained. Dimension 1 explains most of the model and includes variables such as remaining teeth, depression, cognitive decline, number of medications, frailty, age, and education level. Dimension 2 is composed of frequency of sugar intake and uncontrolled blood pressure. Figure 3A depicts clear and distinct patterns of association among variable categories in the MCA, revealing the formation of four clusters based on the number of remaining teeth. These clusters where further analyzed through k-means cluster analysis, identifying some common characteristics shared among the groups. However, two distinct groups stand out: the edentulous group (0 teeth) and the functional dentition group (21 and more teeth). The edentulous group comprises individuals with older age (65 and above), cognitive decline, depression, frailty, and polypharmacy. These characteristics, except for polypharmacy and cognitive decline, are also observed in the second group of patients with severe tooth loss (1–9 remaining teeth). Additionally, in alignment with the Multinomial logistic regression analysis, a noticeable association becomes evident in the fourth group, linking higher educational attainment, the absence of frailty, cognitive decline, depression and uncontrolled blood pressure, lower medication, and lower sugar consumption between meals, with the presence of functional dentition (Figures 3A,B).

## 4 Discussion

This study stands as one of the limited inquiries investigating the oral health condition, treatment requirements, and their interrelation with the socio-medical conditions of older Indigenous individuals in Chile. These objectives are distinctive because they concentrate on an often-overlooked group of older Indigenous individuals who should be assessed independently, rather than being grouped with other cohorts. This distinction is vital because the oral health status and disparities among Indigenous communities often go unnoticed in broader population metrics due to factors such as inadequate representation or constraints stemming from survey methodologies (59). Our findings reveal a significant oral health burden, marked by elevated rates of dental caries, periodontitis, dry mouth, and tooth loss. In addition, there is a substantial load of chronic diseases, encompassing cognitive deterioration, an increased risk of depression, the presence of multiple chronic conditions, and a prevalent state of frailty. This investigation sheds light on the significant disparities in both oral and overall health among Indigenous older adults, influenced by factors such as age, education, and lifestyle.

In examining caries experience, our findings reveal a substantial DMFT index of 26.47 within the 65 to 74 age group, indicating a noteworthy prevalence of dental caries in this demographic. While this index is lower than that reported in a Mapuche-Huilliche community in Chile (28.2) (60) and South American Indigenous groups (28.19) (9), it remains higher than figures from national studies (21.57–25.68) (57, 61–63), a trend that has been consistently observed in previous research (8, 9). Unlike many studies that focus primarily on investigating caries experience, this research provides a broader perspective by delving into the comprehensive oral health status of this community, revealing that health inequalities extend beyond caries to encompass tooth loss, periodontal disease, and xerostomia. Notably, when comparing the functional dentition of the 65–74 age group (5.3%) with figures from national surveys (30.2%) (64), a substantial disparity becomes evident. Likewise, significant discrepancies are observed in the prevalence of periodontitis when compared to Chilean non-Indigenous populations (43.3% vs. 28.8%) (65). While the challenges of periodontal epidemiological comparisons due to divergent case definitions are acknowledged, our study’s outcomes align with those reported in a recent systematic review (7).

Consistent with these findings, the prevalence of dry mouth perception was notably higher (63.2%) than that reported in independent older populations (ranging from 13 to 40%) (66, 67). As for the prevalence of oral mucosal lesions, it is lower than previously reported in cross-sectional national studies (ranging from 34.2 to 53%) (54, 68, 69). Nevertheless, the most prevalent lesion in the participants is consistent with prior findings, as denture stomatitis emerges as the most common condition (54, 69, 70). It is noteworthy that oral potentially malignant disorders comprised 16.67% of all the OML, with a prevalence in our sample of 6.58%, which is higher than the reported in Chilean non-Indigenous populations (1.13–4.7%) (32, 54, 68, 70), and worldwide (4.47%) (71). One possible theory for this could be a greater accumulation of risk factors, such as increased sun exposure due to agricultural activities, local traumatic factors like ill-fitted prosthetics, poor oral health, and dietary elements like maté drinking (72, 73), underscoring the importance of oral health promotion for the prevention of risk factors and oral self-examination in this population. In the context of oral potentially malignant disorders, age could be added as a risk factor, considering that this study focused on older adults (60 years and older), and, according to literature, Leucoplakias, as one of the main potentially malignant disorder are found in patients over 45 years old (74). In the same context, age also could predict probable malignization, considering that epithelial dysplasia is more frequently found in individuals over 41 years old (75). Predictive instruments like nomograms have been developed with the aim of predict malignant transformation of Leucoplakias, concluding that this transformation occurs mainly in patients over 50 years old (76). However, it is essential to approach these results with caution as they were based on clinical diagnosis, and histopathological confirmation is required. To the best of our knowledge, there is no existing evidence on the prevalence of swallowing disorders in Indigenous populations, as they are not routinely identified. The prevalence found in this study is in line with previous global studies, affecting approximately one in every five older individuals (77). Correlations were found with medication consumption, dry mouth, depression and multimorbidity. As the relationship between this geriatric syndrome and oral health conditions remains relatively unexplored (78), it is advisable for future studies to not only include screening but also to broaden their scope, considering its significant impact on health and quality of life. This expansion could encompass clinical evaluations, such as assessing temporomandibular joint disorders (79) and evaluating intra-oral compartment pressures (80).

The current study has identified a robust connection between the number of remaining teeth and several key factors, including age, education, mental and physical health, medication usage, and meal frequency. Individuals with fewer teeth tended to be older, have lower levels of education, experience more mental and physical health issues, consume a greater number of daily medications, and have a higher meal frequency. This observation is consistent with previous research findings in this age group (81–84).

Additionally, it has been reported that as certain Indigenous communities transition from traditional to more contemporary lifestyles, there is a concerning and rapid increase in lifestyle-related illnesses, such as obesity, cardiovascular diseases, and type 2 diabetes (85). Some of the issues highlighted by these findings underscore the importance of health promotion. This is because the primary drivers of complex noncommunicable diseases (NCDs), such as caries, periodontitis, oral cancer, diabetes, hypertension, and others, include an unhealthy diet, reduced physical activity, and tobacco usage (86, 87). Through our investigation, we have uncovered a compelling correlation between treatment requirements and both nutritional status and chronic conditions associated with dietary patterns. One plausible explanation for this phenomenon is the direct impact of the number of remaining teeth on dietary intake quality (88). For instance, a population requiring significant prosthodontic treatment due to tooth loss may have a reduced ability to chew and process certain foods, leading to dietary choices that rely on easily consumable carbohydrates and excessive sugar intake.

The use of technology to deliver care to remote settings has been successful in addressing accessibility barriers for Indigenous older adults (89–91). In this case, the integrated technological ecosystem in TEGO enabled teleconsultation and the provision of specialist guidance to patients. Additionally, it helped reduce the burden on the healthcare system by addressing a large number of low to moderate complexity treatment needs, while also decreasing the need for secondary care consultations, as only those who truly required specialist evaluation had to physically attend a healthcare facility. The integration of electronic records, as exemplified by the TEGO platform, played a pivotal role in enhancing the quality of care provided. This technology, empowered students to perform comprehensive assessments of rural older adults, guiding them in tailoring treatments based on the medical-geriatric status of the patients they evaluated. This community-based intervention also exposed them to the realities of rural practice and service conditions, preparing them to deal with situations they may not have encountered during their training, with remote support from dental specialist to address more complex queries. This approach aligns undergraduate curricula with the health needs of rural communities (92). Furthermore, our study draws parallels with the positive outcomes observed in rural Australia, where a collaborative endeavor between universities and the local community established a student-led clinic service improving access to timely and less invasive care for Indigenous populations (93). These outcomes underscore the potential for technology-driven solutions to bridge healthcare gaps and optimize the delivery of care to underserved communities, including Indigenous older adults in our study.

Considering that oral health disparities persist as a public health concern not only in Chile but also in various regions globally (94–96), this study underscores the necessity for a comprehensive analysis of factors contributing to health inequalities in the studied population. These factors include the historical socioeconomic determinants that have consistently shaped the life trajectories of this specific cohort (97), alongside with the rapid adoption of detrimental lifestyle habits. The study’s identification of a high burden of oral disease, significant unmet oral healthcare needs, particularly in terms of restorative and prosthetic treatment; and the undeniable connection between oral health and overall well-being, highlights the importance of implementing a comprehensive and interdisciplinary healthcare approach, especially when working with Indigenous populations. This approach aligns with the Mapuche perspective on health, seamlessly integrating socio-spiritual and psycho-biological elements into their worldview and environmental interactions (13). However, integrating traditional health concepts faces challenges due to the prevailing specialization in the current healthcare system, which often hinders collaboration and regrettably overlooks the crucial principle of interdependence (98). To effectively address the substantial oral health challenges faced by Indigenous older populations dental public health programs must prioritize culturally appropriate strategies and embrace innovative, community-centered approaches (8).

While considering these observations, it is essential to recognize the constraints inherent in this study. Issues such as the scarcity of information regarding the entire population within this community, the wide dispersion of the population over a large geographical area, challenges in communication, and difficulties in transportation, led to the adoption of convenience sampling method. It has been acknowledged that studies of this kind often encounter challenges in engaging with Indigenous communities (99). Nevertheless, the involvement of an intercultural facilitator and the support from the local council facilitated the engagement of older adults’ community members, especially those who exhibited hesitance in seeking medical and dental care. This approach also enabled the expression of specific concepts in Mapuzugun. The significance of community involvement and consideration of Indigenous perspectives has been previously emphasized (100, 101), and in this case, the program was approved by community leaders but designed and executed without active community participation, highlighting an area of priority for building trust and partnership in future initiatives. It is worth noting that the older individuals who participated were those with the means to travel to the remote care setting, potentially introducing a participation bias. Moreover, this subgroup might display varying levels of disease compared to more susceptible segments, like dependent older adults. Consequently, while this dataset provides valuable insights, it primarily offers preliminary information and should not be construed as indicative of the broader Mapuche older adult population. This is because the distinct cultural and social characteristics of Indigenous peoples hinder the generalizability to other communities. Additionally, this study is constrained by inherent limitations stemming from its cross-sectional design, impeding the establishment of causal relationships, and rendering it incapable of assessing the effectiveness of the employed program. This underscores the necessity for continued investigation in subsequent research efforts and partnership with local communities.

The study introduces an innovative approach to oral healthcare for Indigenous rural older adults, integrating a technological ecosystem and human capital development. This approach provides essential information to update epidemiological profiles of the Mapuche population. The intricate interplay among the presented variables presents a compelling opportunity for national and international healthcare policies to prioritize this group. Efforts should be directed at implementing a comprehensive and integrated horizontal approach of healthcare strategies, supported by technology, aimed at promoting oral health and endorsing the dignified aging of this population. While we believe that existing programs can benefit from the use of technology and home/community-based care, it is crucial to acknowledge the challenges related to technical capacity, structural limitations, and organizational support for an e-health venture in a rural setting, such as internet connectivity, relocation of healthcare personnel and equipment transport, among other factors. For the implementation of these strategies, it is necessary to encompass suitable and culturally competent interventions for oral health education and promotion, preventive, and treatment measures, as well as effective and sustainable financial resource and organizational planning characterized by a long-term view with the aim of preserving both the remaining dentition and supporting structures. These pilot initiatives hold significant potential for improving the oral health of Indigenous populations and, ultimately, for addressing disparities in oral healthcare. Furthermore, there is an urgent need for forthcoming studies to compile regional, national, and international statistics related to Indigenous oral health. Additionally, longitudinal studies are imperative for evaluating the long-term impact of these programs on changes in Indigenous oral health.

## 5 Conclusion

In conclusion, this study provides valuable insights into the oral health and general well-being of older Indigenous adults residing in a rural Mapuche community in Chile. The findings reveal a high burden of oral disease, including high rates of dental caries, periodontal disease, xerostomia, oral mucosal lesions, and tooth loss. Additionally, participants exhibited complex medical-geriatric conditions such as frailty, cognitive decline, depression, polypharmacy, and multiple chronic diseases, all of which were interrelated with tooth loss variables. The implementation of a geriatric technological ecosystem in the community facilitated the resolution of low to moderate complexity pathologies and enabled remote consultations, thereby reducing the necessity for travel to health centers. To address these significant oral health disparities among Indigenous populations, dental public health initiatives could incorporate innovative approaches like teledentistry, as demonstrated in this study. These programs should prioritize oral health education, prevention, and treatment, while fostering partnerships with the communities and considering the unique needs and perspectives of Indigenous populations. The ultimate goal is to promote dignified aging and enhance the overall health and well-being of these communities.

The research group has conducted previous studies regarding patients’ acceptance of this tool. In this sense, the results showed high levels of patient satisfaction after receiving priority or urgent dental care, which reached above 75% in all dimensions of the questionnaire (Access to dental care, user treatment, platform, recommendation) (102).

Although economic costs may vary due to the types of specialty coverage, material costs, and the use of specialized dental equipment, considering the estimated fees for a specialist dentist, the system allows for the optimization of the specialist’s time, enabling them to accommodate their schedule asynchronously within a timeframe that suits them. In this current project, we foresee the participation of a team of specialists in different areas of dentistry, with at least half a day of dedication each week. Within the context of the platform, the service of computer support is required, which in this case is supported by 2 professionals from the Institute of Educational Informatics of our university, plus the server costs to ensure a continuous service without interruption. However, in relation to the need for a stable internet network and the challenging geography of the territory in Chile, it becomes necessary to connect the use of these technologies in the future through satellite internet, in order to create a technological ecosystem that allows for comprehensive coverage of rural territories, which are the ones that unjustly have the least coverage of specialized health services. We believe that the combination of new technologies, like the ones we have fostered with the development of this technological platform, can be applied as teleodontological strategies to enhance coverage for older adults residing in remote geographical areas, respecting their cultural visions, such as the fair access to specialized dental health care in their own territory, also facilitating their travel to major urban centers and improving the interaction between dentists working directly in the communities.

## Data availability statement

The original contributions presented in the study are included in the article/supplementary material, further inquiries can be directed to VB, victor.beltran@ufrontera.cl.

## Ethics statement

The studies involving humans were approved by Universidad de La Frontera Ethics Committee. The studies were conducted in accordance with the local legislation and institutional requirements. Written informed consent for participation in this study was provided by the participants or their legal guardians/next of kin.

## Author contributions

VB: Conceptualization, Funding acquisition, Project administration, Supervision, Writing – review & editing, Investigation, Methodology. FM-S: Conceptualization, Data curation, Investigation, Methodology, Writing – original draft, Writing – review & editing, Visualization. CA: Investigation, Project administration, Writing – review & editing, Data curation, Funding acquisition, Supervision. PN: Formal analysis, Writing – review & editing, Methodology. BV: Writing – review & editing, Investigation, Supervision, Visualization. CS: Investigation, Data curation, Writing – review & editing. PU: Investigation, Data curation, Writing – review & editing. WE: Writing – review & editing, Investigation, Supervision.

## References

[1] Patel J Wallace J Doshi M Gadanya M Ben Yahya I Roseman J. Oral health for healthy ageing. Lancet Healthy Longev. (2021) 2:e521–7. doi: 10.1016/S2666-7568(21)00142-2 36098001

[2] Petersen PE Ogawa H. Promoting Oral health and quality of life of older people - the need for public health action. Oral Health Prev Dent. (2018) 16:113–24. doi: 10.3290/j.ohpd.a40309, PMID: 29736489

[3] World Health Organization (WHO). Decade of healthy ageing. 73rd World Health Assembly Decisions. (2020). Available from: https://www.who.int/news/item/07-08-2020-73rd-world-health-assembly-decisions [Accessed September 15, 2023].

[4] FDI World Dental Federation. FDI policy statement on oral health for healthy ageing: adopted by the FDI general assembly: 24 September 2015, Bangkok, Thailand. Int Dent J. (2016) 66:7–8. doi: 10.1111/idj.12231 26803941 PMC9376517

[5] Petersen PE Bourgeois D Ogawa H Estupinan-Day S Ndiaye C. Policy and practice the global burden of oral diseases and risks to oral health. Bull World Health Organ. (2005) 83:686–93. PMID: 16211157 PMC2626328

[6] Slack-Smith L. Oral health in older indigenous peoples. Gerodontology. (2019) 36:91–1. doi: 10.1111/ger.12417, PMID: 31124190

[7] Nath S Poirier B Ju X Kapellas K Haag D Jamieson L. Periodontal disease inequities among indigenous populations: a systematic review and meta-analysis. J Periodontal Res. (2022) 57:11–29. doi: 10.1111/jre.12942, PMID: 34655251

[8] Nath S Poirier BF Ju X Kapellas K Haag DG Ribeiro Santiago PH. Dental health inequalities among indigenous populations: a systematic review and meta-analysis. Caries Res. (2021) 55:268–87. doi: 10.1159/000516137, PMID: 34107490 PMC8491513

[9] Soares GH Pereira NF Biazevic MGH Braga MM Michel-Crosato E. Dental caries in south American indigenous peoples: a systematic review. Community Dent Oral Epidemiol. (2019) 47:142–52. doi: 10.1111/cdoe.12436, PMID: 30506750

[10] Schuch HS Haag DG Kapellas K Arantes R Peres MA Thomson WM. The magnitude of indigenous and non-indigenous oral health inequalities in Brazil, New Zealand and Australia. Community Dent Oral Epidemiol. (2017) 45:434–41. doi: 10.1111/cdoe.12307, PMID: 28509420

[11] The World Bank. Indigenous Latin America in the twenty-first century. Washington, DC: World Bank Group (2015) Available at: http://documents.worldbank.org/curated/en/145891467991974540/Indigenous-Latin-America-in-the-twenty-first-century-the-first-decade.

[12] Anderson I Robson B Connolly M Al-Yaman F Bjertness E King A. Indigenous and tribal peoples' health (the lancet–Lowitja Institute global collaboration): a population study. Lancet. (2016) 388:131–57. doi: 10.1016/S0140-6736(16)00345-7 27108232

[13] Cuyul A. La política de salud chilena y el pueblo Mapuche. Entre el multiculturalismo y la autonomía mapuche en salud. (2013) [cited 2023. Available from: https://asuntosindigenas.gobiernosantiago.cl/wp-content/uploads/2017/05/La-politica-de-salud-chilena-y-el-pueblo-mapuche.pdf [Accessed Aug 28 2023].

[14] Mejia GC Parker EJ Jamieson LM. An introduction to oral health inequalities among indigenous and non-indigenous populations. Int Dent J. (2010) 60:212–5. doi: 10.1922/IDJ_2565Jamieson04 PMID: 20718305

[15] King M Smith A Gracey M. Indigenous health part 2: the underlying causes of the health gap. Lancet. (2009) 374:76–85. doi: 10.1016/S0140-6736(09)60827-8, PMID: 19577696

[16] Deivanayagam TA English S Hickel J Bonifacio J Guinto RR Hill KX. Envisioning environmental equity: climate change, health, and racial justice. Lancet. (2023) 402:64–78. doi: 10.1016/S0140-6736(23)00919-4, PMID: 37263280 PMC10415673

[17] Ravaghi V Quiñonez C Allison PJ. The magnitude of oral health inequalities in Canada: findings of the Canadian health measures survey. Community Dent Oral Epidemiol. (2013) 41:490–8. doi: 10.1111/cdoe.12043 23383978

[18] Hage E Roo JP Van Offenbeek MAG Boonstra A. Implementation factors and their effect on e-health service adoption in rural communities: a systematic literature review. BMC Health Serv Res. (2013) 13:1–16. doi: 10.1186/1472-6963-13-19 23311452 PMC3575225

[19] Hudson HE. Rural telemedicine: lessons from Alaska for developing regions. Telemed e-Health. (2005) 11:460–7. doi: 10.1089/tmj.2005.11.460, PMID: 16149892

[20] Emami E Harnagea H Shrivastava R Ahmadi M Giraudeau N. Patient satisfaction with e-oral health care in rural and remote settings: a systematic review. Syst Rev. (2022) 11:222–34. doi: 10.1186/s13643-022-02103-2, PMID: 36309732 PMC9617039

[21] Maar MA Seymour A Sanderson B Boesch L. Reaching agreement for an aboriginal e-health research agenda: the aboriginal telehealth knowledge circle consensus method. Rural Remote Health. (2010) 10:1299. doi: 10.22605/RRH1299, PMID: 20108996

[22] Reilly R Stephens J Micklem J Tufanaru C Harfield S Fisher I. Use and uptake of web-based therapeutic interventions amongst indigenous populations in Australia, New Zealand, the United States of America and Canada: a scoping review. Syst Rev. (2020) 9:1–17. doi: 10.1186/s13643-020-01374-x 32475342 PMC7262751

[23] Elliott G Smith AC Bensink ME Brown C Stewart C Perry C. The feasibility of a community-based Mobile telehealth screening Service for Aboriginal and Torres Strait Islander Children in Australia. Telemed e-Health. (2010) 16:950–6. doi: 10.1089/tmj.2010.0045, PMID: 21034237

[24] World Health Organization (WHO). The health of indigenous peoples. (2023) A76/A/CONF./1A76/A/CONF./1: 1–5. Available at: https://apps.who.int/gb/ebwha/pdf_files/WHA76/A76_ACONF1-en.pdf

[25] United Nations. United Nations declaration on the rights of indigenous peoples. Geneva: UN Office of the High Commissioner for Human Rights (OHCHR). (2008).

[26] Pan American Health Organization. Strategy and plan of action on ethnicity and health 2019–2025. Washington, D.C: PAHO (2019).

[27] Gizaw Z Astale T Kassie GM. What improves access to primary healthcare services in rural communities? A systematic review. BMC Primary Care. (2022) 23:313. doi: 10.1186/s12875-022-01919-0, PMID: 36474184 PMC9724256

[28] Instituto Nacional de Estadísticas de Chile (INE). Radiografía de género: Pueblos originarios en Chile 2017. (2018). Available from: https://www.ine.gob.cl/docs/default-source/genero/documentos-de-an%C3%A1lisis/documentos/radiografia-de-genero-pueblos-originarios-chile2017.pdf?sfvrsn=7cecf389_8 [Accessed Oct 16 2023]

[29] Ibacache J Chureo F MCFall S Quidel J. Promoción de la Medicina y Terapias Indígenas en la Atención Primaria de Salud: El Caso de los Mapuche de Makewe-Pelale. Washington, DC: WHO/PAHO (2001).

[30] Plath O. Folklore médico chileno: Antropología y salud. 1st ed. Santiago: Nascimento (1981).

[31] El Poblete M. Pueblo Mapuche: Breve caracterización de su organización social. Santiago; (2019). Available from: https://obtienearchivo.bcn.cl/obtienearchivo?id=repositorio/10221/27459/1/BCN___Poblete___El_Pueblo_Mapuche._Breve_caraterizacion_de_su_organizacion_social_final.pdf. [Accessed Oct 15 2023].

[32] Beltrán V Von Marttens A Acuña-Mardones P Sanzana-Luengo C Rueda-Velásquez SJ Alvarado E. Implementation of a Teledentistry platform for dental emergencies for the elderly in the context of the COVID-19 pandemic in Chile. Biomed Res Int. (2022) 2022:9285. doi: 10.1155/2022/6889285, PMID: 35330690 PMC8938690

[33] Ministerio de Desarrollo Social Gobierno de Chile. Orientaciones al RSH N° 8 Cálculo de la Calificación Socioeconómica. (2019). Available from: http://www.registrosocial.gob.cl/docs/Orientaciones-complementarias-N8_calculo-CSE_VF.pdf [Accessed Oct 15 2023].

[34] Bernaola-Sagardui I. Validación del índice de Barthel en la población española. Enferm Clin. (2018) 28:210–1. doi: 10.1016/j.enfcli.2017.12.001 29397315

[35] World Health Organization. Oral health surveys basic methods. 5th ed. Geneva: World Health Organization. (2013).

[36] Ismail AI Pitts NB Tellez M Banerjee A Deery C Douglas G. The international caries classification and management system (ICCMS™) an example of a caries management pathway. BMC Oral Health. (2015) 15:1–13. doi: 10.1186/1472-6831-15-S1-S9 26391116 PMC4580843

[37] Blesa R Pujol M Aguilar M Santacruz P Bertran-Serra I Hernández G. Clinical validity of the ‘mini-mental state’ for Spanish speaking communities. Neuropsychologia. (2001) 39:1150–7. doi: 10.1016/S0028-3932(01)00055-0, PMID: 11527552

[38] Martínez De La Iglesia J Herrero RD Vilches MCO Taberné CA Colomer CA Luque RL. Cross-cultural adaptation and validation of Pfeiffer’s test (short portable mental status questionnaire [SPMSQ]) to screen cognitive impairment in general population aged 65 or older. Med Clin (Barc). (2001) 117:129–34. doi: 10.1016/s0025-7753(01)72040-4 11472684

[39] Yesavage JA Sheikh JI. Geriatric depression scale (GDS). Clin Gerontol. (1986) 5:165–73. doi: 10.1300/J018v05n01_09

[40] Fried LP Tangen CM Walston J Newman AB Hirsch C Gottdiener J. Frailty in older adults: evidence for a phenotype. J Gerontol. (2001) 56:M146–57. doi: 10.1093/gerona/56.3.M146 11253156

[41] Skou ST Mair FS Fortin M Guthrie B Nunes BP Miranda JJ. Multimorbidity. Nat Rev Dis Primers. (2022) 8:1–22. doi: 10.1038/s41572-022-00376-4 35835758 PMC7613517

[42] Masnoon N Shakib S Kalisch-Ellett L Caughey GE. What is polypharmacy? A systematic review of definitions. BMC Geriatr. (2017) 17:1–10. doi: 10.1186/s12877-017-0621-2 29017448 PMC5635569

[43] Belafsky PC Mouadeb DA Rees CJ Pryor JC Postma GN Allen J. Validity and reliability of the eating assessment tool (EAT-10). Ann Otol Rhinol Laryngol. (2008) 117:919–24. doi: 10.1177/000348940811701210 19140539

[44] Fernández-Rosati J Lera L Fuentes-López E Albala C. Validez y confiabilidad del cuestionario Eating Assessment Tool 10 (EAT-10) para detectar disfagia en adultos mayores chilenos. Rev Med Chile. (2018) 146:1008–15. doi: 10.4067/s0034-98872018000901008, PMID: 30725021

[45] Ismail AI Sohn W Tellez M Amaya A Sen A Hasson H. The international caries detection and assessment system (ICDAS): an integrated system for measuring dental caries. Community Dent Oral Epidemiol. (2007) 35:170–8. doi: 10.1111/j.1600-0528.2007.00347.x, PMID: 17518963

[46] Sociedad Española de Periodoncia y Osteointegración (SEPA). Available at: https://sepa.es/periodontograma/. (2022). Periodontograma.

[47] Botelho J Machado V Proença L Mendes JJ. The 2018 periodontitis case definition improves accuracy performance of full-mouth partial diagnostic protocols. Sci Rep. (2020) 10:1–7. doi: 10.1038/s41598-020-63700-6 32341429 PMC7184582

[48] Chapple ILC Mealey BL Van Dyke TE Bartold PM Dommisch H Eickholz P. Periodontal health and gingival diseases and conditions on an intact and a reduced periodontium: consensus report of workgroup 1 of the 2017 world workshop on the classification of periodontal and Peri-implant diseases and conditions. J Periodontol. (2018) 89:S74–84. doi: 10.1111/jcpe.12940 29926944

[49] Hobdell M Petersen PE Clarkson J Johnson N. Global goals for oral health 2020. Int Dent J. (2003) 53:285–8. doi: 10.1111/j.1875-595X.2003.tb00761.x 14560802

[50] Kassebaum NJ Bernabé E Dahiya M Bhandari B Murray CJL Marcenes W. Global burden of severe tooth loss: a systematic review and meta-analysis. J Dent Res. (2014) 93:20S–8S. doi: 10.1177/0022034514537828, PMID: 24947899 PMC4293725

[51] Beltrán V Acuña-Mardones P Fernández-Gil F Acuña-Mardones D Engelke W. Development of a teledentistry system for the elderly in the context of the COVID-19 pandemic: 3D diagnostic models integration in a web platform. J Oral Res. (2022) 2022:1–7. doi: 10.17126/joralres.2022.024

[52] Venegas B Rueda S Flores M Cerda A Beltrán V. Prevention of Oral cancer through the implementation of a Teledentistry platform for the elderly. Journal of Oral. Demogr Res. (2022) 2022:1–8. doi: 10.17126/joralres.2022.027

[53] Robledo-Sierra J Mattsson U Svedensten T Jontell M. The morbidity of oral mucosal lesions in an adult Swedish population. Med Oral Patol Oral Cir Bucal. (2013) 18:e766–72. doi: 10.4317/medoral.19286, PMID: 23792308 PMC3790650

[54] Espinoza I Rojas R Aranda W Gamonal J. Prevalence of oral mucosal lesions in elderly people in Santiago, Chile. J Oral Pathol Med. (2003) 32:571–5. doi: 10.1034/j.1600-0714.2003.00031.x, PMID: 14632931

[55] Woo SB. Oral pathology: A comprehensive atlas and text. 1st ed. Philadelphia: Elsevier Saunders (2012).

[56] Neville B Damm D Allen C Chi A. Oral and maxillofacial pathology. 4th ed. Missouri: Elsevier (2016).

[57] Mariño R Giacaman RA. Factors related to unmet oral health needs in older adults living in Chile. Arch Gerontol Geriatr. (2014) 58:454–9. doi: 10.1016/j.archger.2014.01.003, PMID: 24556393

[58] Redford M Drury TF Kingman A Brown LJ. Denture use and the technical quality of dental prostheses among persons 18–74 years of age: United States, 1988–1991. J Dent Res. (1996) 75:714–25. doi: 10.1177/002203459607502S11 8594095

[59] Jamieson L Hedges J Peres MA Guarnizo-Herreño CC Bastos JL. Challenges in identifying indigenous peoples in population oral health surveys: a commentary. BMC Oral Health. (2021) 21:1–6. doi: 10.1186/s12903-021-01455-w 33910554 PMC8082663

[60] Angel P Mc F. Cisternas, Moncada G. Prevalencia de caries, Pérdida de Dientes y Necesidad de Tratamiento en Población Adulta Mapuche-Huilliche de Isla Huapi prevalence of caries, teeth loss and treatment needs in adult Mapuche-Huilliche population in Isla Huapi Trabajo de Investigación. Rev Clin Periodoncia Implantol Rehabil Oral. (2010) 3:69–72. doi: 10.1016/S0718-5391(10)70044-6

[61] Urzua I Mendoza C Arteaga O Rodríguez G Cabello R Faleiros S. Dental caries prevalence and tooth loss in chilean adult population: first national dental examination survey. Int J Dent. (2012) 2012:810170:1–6. doi: 10.1155/2012/810170 23316234 PMC3536045

[62] Beltrán V Flores M Sanzana C Muñoz-Sepúlveda F Alvarado E Venegas B. Tooth loss and caries experience of elderly Chileans in the context of the COVID-19 pandemic in five regions of Chile. Int J Environ Res Public Health. (2023) 20:1–11. doi: 10.3390/ijerph20043001, PMID: 36833696 PMC9967189

[63] Quinteros ME Cáceres DD Soto A Mariño RJ Giacaman RA. Caries experience and use of dental services in rural and urban adults and older adults from Central Chile. Int Dent J. (2014) 64:260–8. doi: 10.1111/idj.12118, PMID: 25125265 PMC9376436

[64] Ministerio de Salud (MINSAL). Informe Encuesta Nacional de Salud 2016–2017. Salud Bucal. (2019) Available from: https://goo.gl/oe2iVt. [Accessed Jul 5 2023].

[65] Giacaman RA Sandoval Salas D Bustos Alvarez IP Rojas Cáceres MA Mariño RJ. Epidemiología del estado de salud periodontal en la VII Región del Maule, Chile. Revista Clínica de Periodoncia, Implantología y Rehabilitación Oral. (2016) 9:184–92. doi: 10.1016/j.piro.2016.07.002

[66] Liu B Dion MR Jurasic MM Gibson G Jones JA. Xerostomia and salivary hypofunction in vulnerable elders: prevalence and etiology. Oral Surg Oral Med Oral Pathol Oral Radiol. (2012) 114:52–60. doi: 10.1016/j.oooo.2011.11.014, PMID: 22727092

[67] Ikebe K Nokubi T Sajima H Kobayashi S Hata K Ono T. Perception of dry mouth in a sample of community-dwelling older adults in Japan. Spec Care Dentist. (2001) 21:52–9. doi: 10.1111/j.1754-4505.2001.tb00225.x 11484581

[68] Mariño RJ Cueto A Badenier O Acevedo R Moya R. Oral health status and inequalities among ambulant older adults living in Central Chile. Community Dent Health. (2011) 28:143–8. doi: 10.1922/CDH_2567Marino06 PMID: 21780353

[69] Cueto A Martínez R Niklander S Deichler J Barraza A Esguep A. Prevalence of oral mucosal lesions in an elderly population in the city of Valparaiso. Chile Gerodontology. (2013) 30:201–6. doi: 10.1111/j.1741-2358.2012.00663.x, PMID: 22500979

[70] Lozano C Vergara C Lee X. Prevalence of oral lesions and chronic non-communicable diseases in a sample of Chilean institutionalized versus non-institutionalized elderly. J Oral Res. (2018) 7:108–13. doi: 10.17126/joralres.2018.025

[71] Mello FW Miguel AFP Dutra KL Porporatti AL Warnakulasuriya S Guerra ENS. Prevalence of oral potentially malignant disorders: a systematic review and meta-analysis. J Oral Pathol Med. (2018) 47:633–40. doi: 10.1111/jop.12726 29738071

[72] Kumari P Debta P Dixit A. Oral potentially malignant disorders: Etiology, pathogenesis, and transformation into Oral cancer. Front Pharmacol. (2022) 13:825266. doi: 10.3389/fphar.2022.825266, PMID: 35517828 PMC9065478

[73] Dasanayake AP Silverman AJ Warnakulasuriya S. Maté drinking and oral and oro-pharyngeal cancer: a systematic review and meta-analysis. Oral Oncol. (2010) 46:82–6. doi: 10.1016/j.oraloncology.2009.07.006, PMID: 20036605

[74] Shah JS Dubey J. Prevalence and factors associated with oral potentially malignant disorders and oral squamous cell carcinoma: an institutional study. J Cancer Res Ther. (2023) 19:S536–44. doi: 10.4103/jcrt.jcrt_759_22, PMID: 38384016

[75] Pires FR Barreto ME Nunes JG Carneiro NS Azevedo AB Dos Santos TC. Oral potentially malignant disorders: clinical-pathological study of 684 cases diagnosed in a Brazilian population. Med Oral Patol Oral Cir Bucal. (2020) 25:e84–8. doi: 10.4317/medoral.23197, PMID: 31880285 PMC6982984

[76] Wang T Wang L Yang H Lu H Zhang J Li N. Development and validation of nomogram for prediction of malignant transformation in oral leukoplakia: a large-scale cohort study. J Oral Pathol Med. (2019) 48:491–8. doi: 10.1111/jop.12862 30980769

[77] Rech RS de Goulart BNG dos Santos KW Marcolino MAZ Hilgert JB. Frequency and associated factors for swallowing impairment in community-dwelling older persons: a systematic review and meta-analysis. Aging Clin Exp Res. (2022) 34:2945–61. doi: 10.1007/s40520-022-02258-x, PMID: 36207669

[78] Drancourt N El Osta N Decerle N Hennequin M. Relationship between Oral health status and oropharyngeal dysphagia in older people: a systematic review. Int J Environ Res Public Health. (2022) 19:3618. doi: 10.3390/ijerph192013618 36294196 PMC9602827

[79] Gilheaney Ó Béchet S Kerr P Kenny C Smith S Kouider R. The prevalence of oral stage dysphagia in adults presenting with temporomandibular disorders: a systematic review and meta-analysis. Acta Odontol Scand. (2018) 76:448–58. doi: 10.1080/00016357.2018.1424936, PMID: 29320883

[80] Engelke W Jung K Knösel M. Intra-oral compartment pressures: a biofunctional model and experimental measurements under different conditions of posture. Clin Oral Investig. (2011) 15:165–76. doi: 10.1007/s00784-009-0367-0, PMID: 20127264 PMC3056003

[81] Cannon I Robinson-Barella A McLellan G Ramsay SE. From drugs to dry mouth: a systematic review exploring Oral and psychological health conditions associated with dry mouth in older adults with polypharmacy. Drugs Aging. (2023) 40:307–16. doi: 10.1007/s40266-023-01017-5, PMID: 36943673

[82] Cademartori MG Gastal MT Nascimento GG Demarco FF Corrêa MB. Is depression associated with oral health outcomes in adults and elders? A systematic review and meta-analysis. Clin Oral Investig. (2018) 22:2685–702. doi: 10.1007/s00784-018-2611-y, PMID: 30191327

[83] Xu K Yu W Li Y Li Y Wan Q Chen L. Association between tooth loss and hypertension: a systematic review and meta-analysis. J Dent. (2022) 123:104178. doi: 10.1016/j.jdent.2022.104178, PMID: 35661800

[84] Gao C Larvin H Bishop DT Bunce D Pavitt S Wu J. Oral diseases are associated with cognitive function in adults over 60 years old. Oral Dis. (2023):1–9. doi: 10.1111/odi.14757 37811600

[85] Gracey M King M. Indigenous health part 1: determinants and disease patterns. Lancet. (2009) 374:65–75. doi: 10.1016/S0140-6736(09)60914-4 19577695

[86] Chapple ILC Bouchard P Cagetti MG Campus G Carra MC Cocco F. Interaction of lifestyle, behaviour or systemic diseases with dental caries and periodontal diseases: consensus report of group 2 of the joint EFP/ORCA workshop on the boundaries between caries and periodontal diseases. J Clin Periodontol. (2017) 44:S39–51. doi: 10.1111/jcpe.12685 28266114

[87] World Health Organization. Global oral health status report: towards universal health coverage for oral health by 2030. (2022). Available from: http://apps.who.int/bookorders. [Accessed Oct 15 2023].

[88] Zhu Y Hollis JH. Tooth loss and its association with dietary intake and diet quality in American adults. J Dent. (2014) 42:1428–35. doi: 10.1016/j.jdent.2014.08.012, PMID: 25174947

[89] Poprawski DM Adams J Bassal A. P0225 telemedicine: a novel approach of bringing oncology care closer to the patient. Eur J Cancer. (2014) 50:e72. doi: 10.1016/j.ejca.2014.03.269

[90] Jin AJ Martin D Maberley D Dawson KG Seccombe DW Beattie J. Evaluation of a mobile diabetes care telemedicine clinic serving aboriginal communities in northern British Columbia. Canada Int J Circumpolar Health. (2004) 63:124–8. doi: 10.3402/ijch.v63i0.17871, PMID: 15736635

[91] Choukou MA Maddahi A Polyvyana A Monnin C. Digital health technology for indigenous older adults: a scoping review. Int J Med Inform. (2021) 148:104408. doi: 10.1016/j.ijmedinf.2021.104408 33609927

[92] Strasser R Kam SM Regalado SM. Rural health care access and policy in developing countries. Annu Rev Public Health. (2016) 37:395–412. doi: 10.1146/annurev-publhealth-032315-021507 26735432

[93] March S Mangoyana C Oakley P Lalloo R Walsh LJ. Positive impacts of oral health services provision by a student-led primary care clinic to an Australian rural indigenous community. Aust Dent J. (2023) 68:151–9. doi: 10.1111/adj.12960, PMID: 37150594

[94] Peres MA Macpherson LMD Weyant RJ Daly B Venturelli R Mathur MR. Oral diseases: a global public health challenge. Lancet. (2019) 394:249–60. doi: 10.1016/S0140-6736(19)31146-8 31327369

[95] Elani HW Harper S Thomson WM Espinoza IL Mejia GC Ju X. Social inequalities in tooth loss: a multinational comparison. Community Dent Oral Epidemiol. (2017) 45:266–74. doi: 10.1111/cdoe.12285, PMID: 28185272

[96] Borgeat Meza M Espinoza I Carvajal P Cuevas R. Changes in oral health inequalities in adults in Chile. Community Dent Oral Epidemiol. (2022) 50:506–12. doi: 10.1111/cdoe.12701, PMID: 34713473

[97] Jamieson LM Elani H Mejia GC Ju X Kawachi I Harper S. Inequalities in indigenous oral health: findings from Australia, New Zealand, and Canada. J Dent Res. (2016) 95:1375–80. doi: 10.1177/0022034516658233, PMID: 27445131

[98] Stange KC. The problem of fragmentation and the need for integrative solutions. Ann Fam Med. (2009) 7:100–3. doi: 10.1370/afm.971, PMID: 19273863 PMC2653966

[99] Tsai C Blinkhorn A Irving M. Oral health programmes in indigenous communities worldwide—lessons learned from the field: a qualitative systematic review. Community Dent Oral Epidemiol. (2017) 45:389–97. doi: 10.1111/cdoe.12302, PMID: 28425612

[100] McIlduff CD Acharibasam J Starr V Chapados M. Engaging indigenous older adults with technology use to respond to health and well-being concerns and needs. Healthc Manage Forum. (2022) 35:257–64. doi: 10.1177/08404704221103521, PMID: 35670368 PMC9425719

[101] Moecke DP Holyk T Beckett M Chopra S Petlitsyna P Girt M. Scoping review of telehealth use by indigenous populations from Australia, Canada, New Zealand, and the United States. J Telemed Telecare. (2023):1–19. doi: 10.1177/1357633X231158835 PMC11411853 36911983

[102] Beltrán V Acuña-Mardones P Díaz J Alvarado E von Marttens A. TEGO: a new concept of teledentistry for the elderly through a web platform and mobile app in the context of the covid-19 pandemic. J Oral Res. (2022) S:1–8. doi: 10.17126/joralres.2022.023

